# Temporally Unconstrained Decoding Reveals Consistent but Time-Varying Stages of Stimulus Processing

**DOI:** 10.1093/cercor/bhy290

**Published:** 2018-12-07

**Authors:** Diego Vidaurre, Nicholas E Myers, Mark Stokes, Anna C Nobre, Mark W Woolrich

**Affiliations:** Department of Psychiatry, Oxford Centre for Human Brain Activity (OHBA), University of Oxford, Oxford, UK

**Keywords:** between-trial temporal variability, decoding analysis, representational states, sequential stimulus processing

## Abstract

In this article, we propose a method to track trial-specific neural dynamics of stimulus processing and decision making with high temporal precision. By applying this novel method to a perceptual template-matching task, we tracked representational brain states associated with the cascade of neural processing, from early sensory areas to higher order areas that are involved in integration and decision making. We address a major limitation of the traditional decoding approach: that it relies on consistent timing of these processes over trials. Using a TUDA approach, we found that the timing of the cognitive processes involved in perceptual judgments can vary considerably over trials. This revealed that the sequence of processing states was consistent for all subjects and trials, even when the timing of these states varied. Furthermore, we found that the specific timing of states on each trial was related to the quality of performance over trials. Altogether, this work not only highlights the serious pitfalls and misleading interpretations that result from assuming stimulus processing to be synchronous across trials but can also open important avenues to investigate learning and quantify plasticity.

## Introduction

Neural processing of a stimulus and its use in guiding behavior are highly dynamic. A given stimulus typically elicits a cascade of activation across the brain, including its multiple parallel as well as re-entrant pathways, starting with early feature analysis and leading to increasing integration and decision making (see, e.g., Meyers et al. 2008; Harvey et al. 2012), but, to which extent is it possible to capture the progressive stages of this information-processing cascade related to stimulus processing from non-invasive human brain imaging data?

An influential approach has been to use decoding models that capture how the current stimulus is represented in the brain activity (Haynes and Rees 2006; Norman et al. 2006; Tong and Pratte 2012; Haxby et al. 2014; Grootswagers et al. 2017). Assuming we have a number of trials or repetitions of a certain process (e.g., the presentation of a stimulus), the standard approach for decoding is to separately train one classifier or regression model (depending on whether the stimulus is categorical or continuous) at each time point, by pooling together the data from all trials in the training set and then testing for the accuracy of each of these models on a test dataset (King and Dehaene 2014). This can then be used to interrogate the temporal dynamics of the processes evoked by the stimulus (see, e.g., Meyers et al. 2008; Carlson et al. 2011; Carlson et al. 2013; Stokes et al. 2013; Isik et al. 2013; King et al. 2014). However, this approach is, by construction, based on the assumption that these brain processes are “synchronous” across trials, that is, the different stages of information processing start and finish at the same time within each trial.

Here, we argue that assuming consistent timing over trials may be too restrictive, ignoring trial-to-trial variability in the dynamics. Furthermore, it can severely misrepresent the data by leading to a potentially false conclusion of persistent activity, artefactually created by the act of averaging across trials (Latimer et al. 2015; Lundqvist et al. 2016; Stokes and Spaak 2016). Sometimes, this assumption can also induce the impression of having a high number and relatively rapid succession of distinct processing states, as a consequence of trial-to-trial variability in the onset and duration of a potentially much smaller succession of states. Such temporal variability of processing states could be ubiquitous. For example, different stages of information processing may start and finish at different time points in each trial, depending on different levels of arousal or selective attention at the time of stimulus onset, or as a result of learning and plasticity.

In this article, we propose a new probabilistic (Bayesian) framework for identifying representational brain states with high temporal resolution and with no assumption about the states having to occur at fixed time points on each trial. We refer to it as temporally unconstrained decoding analysis (TUDA). In this approach, as opposed to previous work where states are purely defined in terms of brain activity patterns with no information of the stimulus (Vidaurre et al. 2016, 2018; Vidaurre, Abeysuriya, et al. 2017; Vidaurre, Smith, et al. 2017), a representational state is defined here as a functional decoding model that characterizes a relation between brain activity and the current stimulus (Haynes and Rees 2006). Each state is distinct from other states not just in their brain activity fingerprint but, specifically, in describing how and in which regions the brain represents the stimulus, such that a switch of state indicates that the decoding does not cross-generalize before and after the switch (i.e., the stimulus-specific pattern of activity has changed). TUDA thus estimates the decoding weights associated to each decoding model and identifies when each decoding model is “active” for each trial. Crucially, this is done without restricting the model to be active at the same point in time on each trial.

By applying this approach to magnetoencephalography (MEG) recordings in a perceptual judgment task, we found that, when allowing for this temporal flexibility, a reduced number of decoding models (fewer than six) is sufficient to explain the between-trial temporal differences in the data. This compares with standard decoding, which, with one model per within-trial time point (typically more than 100), cannot access this information at all. Furthermore, we found that the temporal dynamics of the decoding models correlate with behavioral changes over trials, lending additional support to the physiological relevance of between-trial temporal variability of the underlying neural processing cascade. To be able to meaningfully relate this information to behavior is not only useful but also proves the existence of tangible and interpretable differences in stimulus processing between trials.

## Methods

### Task and Participants 

This study used previously published data used for a different purpose (described in Myers et al. 2015). Ethical approval for methods and procedures was obtained from the Central University Research Ethics Committee of the University of Oxford. In brief, we recorded MEG data while participants performed a template-matching visual task (EEG data were also simultaneously acquired but were not used in the current study). Ten right-handed volunteers (age range: 21–27 years, six females) took part in the study, completing two sessions each, containing short blocks. In each block, participants were presented with one orientation template to keep in mind. They then viewed a stream of oriented gratings and responded when the presented angle matched the template angle.

The task consisted of eight brief (approximately 6 min) blocks, in which 480 stimuli were presented (resulting in a total of 3840 stimulus presentations per session). Each block began with the presentation of a target orientation (drawn at random, without replacement, from the 16 stimulus orientations), displayed centrally as a green line (4° length). The stimulus stream consisted of randomly oriented Gabor patches, presented centrally for 100 ms, at an average rate of 650 ms. Stimuli had 16 possible angles (5.625–174.375°, in steps of 11.25°). Participants were instructed to respond whenever a Gabor patch with a matching orientation appeared. Since stimuli were drawn uniformly from the 16 possible orientations, 1/16 of all stimuli were targets. The angles were encoded into two covariates using the sine and cosine functions. Each block was cut into three shorter segments, giving participants brief rest periods. During the rest periods, the target orientation was presented again as a reminder. Participants were instructed to respond as quickly and accurately as possible.

### MEG Data Acquisition and Preprocessing 

Neuromagnetic data were acquired using a whole-head VectorView system (204 planar gradiometers, 102 magnetometers; Elekta Neuromag). The signals were sampled at a rate of 1000 Hz and online band-pass filtered between 0.03 and 300 Hz. Data were preprocessed using the OSL software library (https://ohba-analysis.github.io/osl-docs, last accessed on 13 November, 2018). The raw MEG data were visually inspected for artefacts, de-noised and motion-corrected using Maxfilter Signal Space Separation (Taulu et al. 2004) and downsampled to 250 Hz. Artefacts arising from eye blinks and heartbeats were removed via independent component analysis. Epochs were generated around each stimulus onset (from 0 to 0.6 s) and visually inspected to eliminate any remaining trials with excessive noise.

### Standard Decoding Analysis

**Figure 1.**
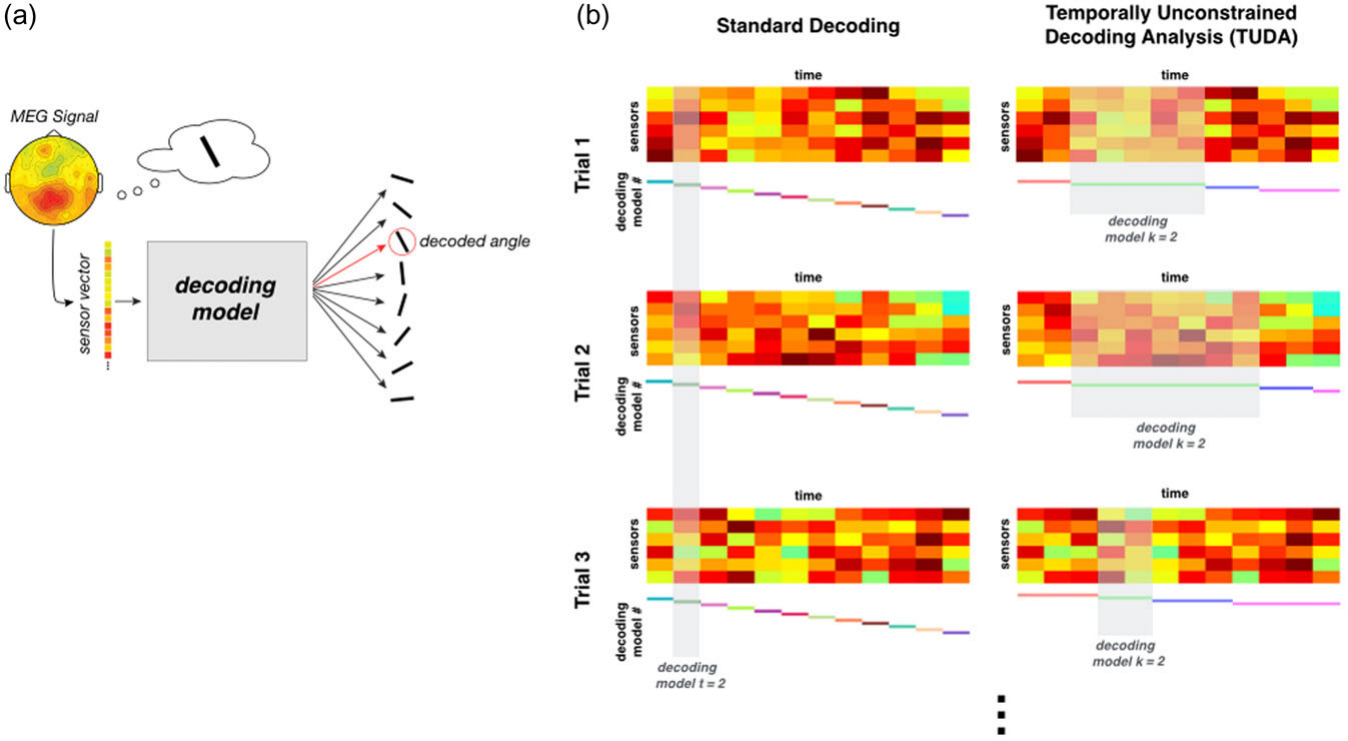
(*a*) General representation of decoding analysis. (*b*) A schematic representation of the standard decoding approach (left) versus the TUD (right).

The standard approach for decoding, illustrated in Figure 1*a*, estimates one decoding model at each time point. We will assume for simplicity that the stimulus is a continuous variable, such that a decoding model is a regression model (in this study, the stimulus is represented by two continuous variables or features: sine and cosine of the corresponding angle). For a categorical variable (e.g., which class of stimulus is being presented), the equations below can be easily adapted to use, for instance, logistic regression. Further extensions using more complex estimation methods (support vector machines, neural networks, etc.) are possible if they are formulated within the Bayesian paradigm.

We now summarize our approach; the notation presented here and in the next sections is summarized in Table 1. Let *t* = 1, …, *T* index time, let *X*_*t*_ be a (trials by channels) matrix containing the data at *t* and let *Y*_*t*_ be a (trials by stimulus features) vector containing the stimulus. The solution for the decoding model at *t* is computed as

(1) $$Y_{t}=X_{t}v_{t}+\varepsilon_{t},$$

where *ε*_*t*_ is Gaussian-distributed noise and *v*_*t*_ is a (channels by stimuli) matrix of decoding weights. Therefore, *v*_*t*_ represents the encoding model that predicts the response as a function of the data, for time point *t*. Given this, *v*_*t*_ is typically obtained by maximum likelihood as

(2) $$v_{t}={\left({X\prime }_{t}\;X_{t}\right)}^{-1}\;{X\prime }_{t}\;Y_{t},$$

where ′ represents matrix transposition. In this case, we decode the pair of variables formed by the sine and cosine of the angle of interest, such that *Y*_*t*_ has two columns. In this paper, also, the data was projected into 48 principal components (explaining on average 96% of the variance in the data) for computational reasons, such that *v*_*t*_ has dimension (48 by 2).

**Table 1:** Notation used for the standard decoding approach and TUDA

Notation	Meaning		Meaning
*T*	Time points per trial	*m*_*k*_	*k*th Decoding model
*N*	Number of trials	*w*_*k*_	Decoding weights of *m*_*k*_
*K*	Number of decoding models	*γ*_*stk*_	Probability of *m*_*k*_ to be active at *s* and *t*
*s, t* and *l*	Indexes for trials, time and sensors	*Θ*_*l,k*_	Prob. of going from state *l* to *k*
*Y*_*st*_	Stimulus at time point *t* and trial *s*	*π*_*k*_	Initial prob. For state *k*
*X*_*st*_	Data at time point *t* and trial *s*	*e*_*tj*_	Error of model *v*_*t*_ at time *j*
*v*_*t*_	Standard approach decoding weights	*D*	Between models divergences
*ε*_*st*_	Gaussian-distributed noise	*b*_*lk*_	Encoding model weights

### Temporally Unconstrained Decoding Analysis 

We introduce a novel probabilistic model leaning on the principles of the Hidden Markov model (HMM; Rabiner 1989; Vidaurre, Abeysuriya, et al. 2017; Vidaurre, Smith, et al. 2017; Vidaurre et al. 2018). The basic principle of the HMM is that it represents the data using a discrete number of states. Each state is defined as an instantiation of a certain family of probability distributions—for example a Gaussian distribution (Vidaurre, Smith, et al. 2017). Given such a distribution, the definition of each state is therefore given by a specific set of parameters: in the case of the Gaussian distribution, a mean vector and a covariance matrix. Together with these parameters, the HMM inference estimates the probability of each state being active at each time point and also the probability of transiting from one state to another. These three elements—state distributions, state probabilities at each time point and transition probability matrix—are jointly estimated from the data. The model introduced in this work, TUDA, is based on these basic principles, with the additional new idea that each state is a decoding model. Therefore, different states represent different stages of stimulus processing and the activation and deactivation of the different states mark the progression of this processing through the different stages in the neural hierarchy. We next present the model in detail.

The TUDA model contains each of the decoding models and the probability of each decoding model to be active at each time point at any given trial, together with the transition probability matrix (containing the probability of transitioning from one decoding model to another within the trials). The difference with the standard HMM is then that the states explicitly model the relation between brain activity and the stimulus, that is, each state is a decoder and what determines a change in state is “how” the stimulus is decoded from the data. The difference with the standard decoding approach is that (rather than assuming synchronicity between trials) TUDA deals with between-trial temporal variability by allowing decoders to have specific time courses (i.e., when these become active) for each trial, therefore having the potential to allow different trials to be described by different decoders at a given common time point.

The basic functioning of the TUDA approach is illustrated in Figure 1*b* (right) (see Table 1 for a summary of notation). Let us refer to each decoding model as *m*_*k*_ and assume that the data can be reasonably described with *K* models. Each decoding model *m*_*k*_ is parametrized by a (channels by stimulus features) matrix of decoding weights, denoted as *w*_*k*_. Here, *w*_*k*_ has the same dimensions than *v*_*t*_ (see eq. 1), the only difference being that instead of having a matrix of decoding weights per time point, we now have a matrix of decoding weights per state. As before, then, these parameters represent a linear mapping from the data to the stimulus, that is, they predict the stimulus using the data. (Again, the data were projected in this particular case into 48 principal components and the number of stimulus features is two, such that *w*_*k*_ has in this case dimension 48 by 2.) Therefore, we are reducing the model complexity from having *T* different decoding models to have only *K* decoding models. In mathematical terms, given time point *t* and trial *s*, let us define *Y*_*st*_ as the (1 by stimulus variables) value of the stimulus and *X*_*st*_ as the (one by channels or PCA components) data vector. The top-level model in this case is formulated as

(3) $$Y_{st}=X_{st}(\Sigma_{k}\;\gamma_{stk}\;w_{k})+\varepsilon_{st},$$

where *γ*_*stk*_ = Pr_*st*_*(m*_*k*_) is the probability of model *k* being active at time point *t* and trial *s* and *ε*_*st*_ is the Gaussian-distributed noise at time point *t* and trial *s* (with zero mean and a shared variance parameter that is not model dependent). Here, the decoding weights *v*_*t*_ from equation (1), corresponding to the standard decoding approach, are replaced by *Σ*_*k*_*γ*_*stk*_*w*_*k*_. This allows for the use of a potentially different set of decoding weights *w*_*k*_ at time *t* in each trial *s*, in contrast to the standard decoding approach where the same decoding weights at time *t* are used for all trials. Specifically, the between-trial temporal flexibility is represented by the fact that, as opposed to the standard decoding approach, the probability of model *k* being active at time point *t*, *γ*_*stk*_, is different in each trial; this is illustrated in the right panel of Figure 1*b*, where *γ*_*stk*_ is represented by the gray boxes (although, note that during the inference, *γ*_*stk*_ is not a hard assignment but a probability). Hence, whereas the decoding weights *w*_*k*_ are defined at the group level (i.e., they are common for all trials), the decoding temporal patterns are, crucially, allowed to be trial specific.

We model the probability of transition between decoding models as

(4) $$\gamma_{stk}=Pr_{st}\left(m_{k}\right)=\Sigma_{l}\;\Theta_{l,k}\;Pr_{s,t-1}\left(m_{l}\right)$$

This way, the estimated probability for the decoding model *m*_*k*_ to be active at some time point depends not only on its decoding performance at each specific time point (eq. 3) but also on the decoding model that was active in the previous instant for this trial (eq. 4, which reflects the Markovian property of the HMM). Together with the transition probabilities *Θ*_*l,k*_, we also model the initial model probabilities *π*_*k*_, referring to which is the model active at the start of the trials. As explained next, all these elements are estimated together from the data.

### Inference of the Parameters 

Given the data, we need to estimate the decoding weights *w*_*k*_, the probabilities *γ*_*stk*_, the transition probabilities *Θ*_*l,k*_ and the initial probabilities *π*_*k*_. After describing the approach conceptually in the last section, we now describe in detail the estimation algorithm, which is also illustrated in Supplementary Figure 1. We perform the estimation in two separate steps: first, the decoding weights *w*_*k*_ (Supplementary Fig. 1*a*–*c*) and then the other parameters (Supplementary Fig. 1*d*). Although it is possible to perform the estimation of all parameters simultaneously, we adopt this strategy so that the results are comparable to the standard approach (see below).

In order to estimate the state decoding weights *w*_*k*_, we take a start with the standard decoding approach described above by estimating one decoding model *v*_*t*_ at each time point (Supplementary Fig. 1*a*); that is, at this stage we impose between-trial synchronicity by pooling all trials all pooled together at each time point. We then compute the error of each model *v*_*t*_ for each time point *j* as

(5) $$e_{tj}=\Sigma_{s}{\left(X_{sj}\;v_{t}–Y_{sj}\right)}^{2}$$

In words, *e*_*tj*_ is the across-trials error of model *v*_*t*_ evaluated at time point *j*. Therefore, for each pair of time points (*t,j*), a measure of divergence between the corresponding models *v*_*t*_ and *v*_*j*_ can be then obtained as

(6) $$D_{tj}=e_{tj}+e_{jt}.$$

The resulting matrix *D*, exemplified in Supplementary Figure 1*b* (bottom), is analogous to the across-time generalization matrix often used in the field (King and Dehaene 2014). Based on the matrix *D*, with elements as defined in equation (6), we use hierarchical clustering to group the *T* decoding models *v*_1_, *…*, *v*_*T*_ into *K* clusters; the representatives of these clusters constitute our first approximation to the decoding weights *w*_*k*_ (Supplementary Fig. 1*b*, top). Importantly, these models are based on the standard approach and are constrained to be synchronous across trials, that is, at a given time point the same decoding model is active for all trials.

Still keeping the between-trial synchronicity restriction, we then refined the estimation of *w*_*k*_ by using the expectation–maximization (EM) algorithm (Bishop 2006), where we alternatively estimate *w*_*k*_ and the time points when *w*_*k*_ is active; the EM algorithm is initialized with the cluster representatives from the previous step (Supplementary Fig. 1*c*). At this stage, we stress, if *w*_*k*_ is active at time point *t* that means that it is active for all trials at time point *t*. In Results, this is what we refer to as “having *K* instead of *T* decoding models, while still restricting the decoding models to be synchronous.” The final parameters *w*_*k*_ correspond to the output of this step, which is also passed on to the next step.

In the last step, then, we fix the estimation of *w*_*k*_ to the previous estimation and proceed to dispense with the between-trial synchrony restriction. For this, we use a fully Bayesian approach, estimating the a posteriori distribution of *γ*_*stk*_ and *Θ*_*l,k*_ using variational inference (Supplementary Fig. 1*d*; Wainwright and Jordan 2008). The estimation of the probabilities of each state *k* being active at each time point *t* and trial *s* (i.e., *γ*_*stk*_) is performed using the HMM forward–backward equations. In summary, the forward–backward equations, described in detail elsewhere (Rabiner 1989; Vidaurre et al. 2016), provide a recursive estimation of these probabilities based on how well each state *m*_*k*_ describes the data at time point *t*, the estimated probabilities at time point *t* − 1 and the transition probabilities *Θ*_*l,k*_. In contrast, the computation of the transition probabilities *Θ*_*l,k*_ is trivial given the current estimation of the probabilities *γ*_*stk*_ (Rabiner 1989). These two estimations, of *γ*_*stk*_ and of *Θ*_*l,k*_, are repeated until convergence.

For the last Bayesian variational inference step (Supplementary Fig. 1*d*), we make use of the functions from the HMM–MAR model (where MAR stands for multivariate autoregressive model; Vidaurre et al. 2016) implemented in the toolbox (https://github.com/OHBA-analysis/HMM-MAR). This corresponds to reparameterising the required equation for each decoding model into a mathematically equivalent MAR model.

### Predicting Reaction Time 

Unlike the information of the stimulus itself, the information of reaction time (RT) was not included in the model. That allowed us to use the relation of such model time courses to RT to add further confidence on the biological relevance of the estimated models. For this, we used model time courses to predict RT, discarding trials without a button press. More specifically, we used principal component analysis to reduce the regressor dimensionality from *T* (number of time points) per trial to 25 principal components. This was done for each decoding model separately. We then used sparse regularized regression (Vidaurre et al. 2013) where the regularization parameter was itself chosen using cross-validation.

### Encoding Models 

As discussed in Weichwald et al. (2015), interpreting the magnitude of the decoding weights *w*_*k*_ is not straightforward. This is not specific to TUDA or any other decoding algorithm but applies to every approach that aims at predicting the stimulus using brain data. For example, if the regression coefficient of one sensor has twice as magnitude than another sensor, we cannot infer that the former’s signal is twice as important for stimulus processing; or, if the regression coefficient of one sensor is zero (or almost zero), that does not mean that this sensor is unrelated to how the stimulus is processed. As shown in detail by Weichwald et al. (2015), causal interpretation of spatial coefficients is only possible for encoding models (i.e., those that predict each voxel using the stimulus as a regressor). Therefore, if we wish to examine the spatial extents of the regions involved in stimulus processing, we need to resort to encoding models as in (Myers et al. 2015). That is, instead of using weights that predict the stimulus using the data from the entire sensor space, we construct spatial maps using the encoding weights *b*_*lk*_ that, from the stimulus, predict the data separately at each sensor *l* (i.e., the prediction goes in the opposite direction). Fortunately, by using the decoding model time courses *γ*_*stk*_, it is straightforward to associate a set of encoding weights *b*_*lk*_ to each decoding weights matrix *w*_*k*_. The encoding weights are computed as

(7) $$b_{lk}={\left(Y\prime \;diag\left(\gamma_{k}\right)\;Y\right)}^{-1}\;\left(Y\prime \;diag\left(\gamma_{k}\right)X^{l}\right),$$

where *X*^*l*^ represents the concatenated data for sensor *l*, *γ*_*k*_ are the concatenated decoding model time courses for state *k* and diag() diagonalises the vector argument into a diagonal matrix. In this case, *b*_*lk*_ has two elements because *Y* has two columns (sine and cosine of the corresponding angle). We finally use the *b*_*lk*_ weights to obtain the explained variance per sensor as shown in Figure 4.

### Cross-Validation 

One important question investigated through decoding is when (and where) the brain is processing the stimulus. This is typically addressed by using cross-validation, where, for each cross-validation fold, we estimate a decoding model at each time point using the training trials and test each of the models in the held-out trials at each time point. For TUDA, as discussed earlie, this is problematic because the temporal information of the decoding models in the held-out trials is in principle unknown (it uses the stimulus), so we do not know which model to use in the held-out data without having the stimulus. If we are to use cross-validation to compare different models (for instance, the standard decoding approach and TUDA), either we cannot use the stimulus information, or else we need to correct for the bias brought about by using the model time courses in the held-out trials. Here we do both and correct the introduced bias by the use of surrogate data, as detailed next. (Note that these considerations do not apply for the RT prediction, which was not included into the model in the first place.)

Cross-validation is then carried out in two different ways:

- In an unbiased way, by using, in each held-out trial, the decoding model that is most active on average at each time point in the training trials (therefore losing this temporal variability).
- In a biased way, by using, in each held-out trial, the best combination of decoding models found by TUDA at each time point (this maintains temporal variability but is biased as it is using the stimulus information for each held-out trial to determine the best combination of decoding models to use at each time point).

We generated random samples (surrogates) of the dataset by permuting the labels (here, the angle values) across trials. Using the estimated model time courses (which used data and stimulus), we then run cross-validation on each surrogate dataset to assess *w*_*k*_. This gave us a measure of accuracy per surrogate, which we used to compute the 5% percentile of accuracy across surrogates. These provided, for each cross-validation approach, a baseline that we can then subtract from the original cross-validation estimates. Since neither the surrogates nor the original data have an unbiased assessment of accuracy, this procedure is able to correct for any introduced bias (i.e., even if we use the “biased way” for cross-validation above). For the sake of comparison, we also corrected the unbiased estimations of accuracy in this way. We refer to the result as adjusted CV-*R*^*2*^. Since we are now comparing differences to the surrogate accuracies, overfitting gets accounted for and we can then compare between different approaches including the standard decoding.

## Results

We have proposed a new method, TUDA, which finds decoding models, or states, that specifically characterize how the current stimulus is represented in the brain at different points in time. Crucially, TUDA estimates the exact timing of the decoding models (i.e., at which time points each decoding model activates) together with the regression weights that constitute each decoding model. This approach requires many fewer parameters to predict the stimulus from the data than the standard decoding approach, which assumes consistent timing over trials and fits one model per time point (Fig. 1*b***)**. In exchange, TUDA adds a new degree of freedom: that is, “when” each decoding model is active in each trial, which is inferred in a data-driven manner.

First, we demonstrate on synthetic data the interpretation caveats of the standard decoding approach when neural processing across trials is not synchronous. Then, we apply TUDA to (real) sensor space MEG data collected when subjects were performing a perceptual judgment task, where we show the interpretation advantages of TUDA in practice.

### Standard Decoding Misrepresents the Data If Trials Are Not Synchronous 

Using the standard approach for decoding, it has often been observed that the decoding models (e.g., the regression weights) continuously fluctuate as a function of time. This sometimes leads to poor decoding generalization across time—that is, a model trained at one time point has low accuracy when tested at a different time point (King and Dehaene 2014). If we consider such regression weights as a proxy of the underlying neural processes that are relevant to the task, it can be thus inferred that such brain processes are highly dynamic (see, e.g., Meyers et al. 2008; Carlson et al. 2011, 2013; Stokes et al. 2013; Isik et al. 2013; King et al. 2014); that is, the brain goes through a large number of different processing states that are not necessarily interchangeable. Here, we argue that this can potentially be caused in an artefactual manner by the temporal variability between trials. In order to show how standard decoding can misrepresent the data when stimulus processing is not synchronous across trials, we tested this approach on synthetic data. In this scenario, we generated synthetic data using five decoding models or states (each, a linear function mapping data to stimulus) that cycle through during the trial sequentially.

More specifically, we sampled *N* = 200 trials of two second duration, assuming sampling frequency of 500 Hz (i.e., 1000 time points per trial). The stimulus is modeled to be a color, with three features representing Red-Green-Blue (RGB) coordinates. For each trial, the color stimulus was randomly sampled from a three-dimensional uniform distribution (with values between zero and one). The data are set to have twenty channels. We assume there are five “cognitive processes” (or ground-truth states) underlying stimulus processing. These can be regarded as the different stages of stimulus processing across the anatomical hierarchy. Each of these is modeled as a (three RGB coordinates by 20 channels) matrix of coefficients. At each time point and trial, the data are generated by multiplying the current color (one time point by three RGB coordinates) by the corresponding matrix of coefficients (three RGB coordinates by 20 channels) and then adding some Gaussian noise (standard deviation equal to 0.1). We set each of the ground-truth states to activate or deactivate a different subset of the channels (four channels per state). More specifically, for each state, we sample the (three RGB coordinates by four channels) active coefficients from a uniform distribution and the rest are set to zero. All trials are set to start in state 1, and transitions are always from state 1 to state 2, from state 2 to state 3 and from state 3 to state 4 and then to state 5. The average duration of the state visits is set to be, respectively, for each state, 0.2, 0.3, 0.4, 0.5 and 0.6 s. The actual dwell time of each state visit is modelled using a Gaussian distribution with variances 0.02, 0.05, 0.12, 0.5 and 0.8 s. This is sampled for each trial separately, so that the time courses of the decoding models are slightly different across trials. As a result of this, the between-trial variability, regarding which model is active, increases as we progress through the trial.

**Figure 2.**
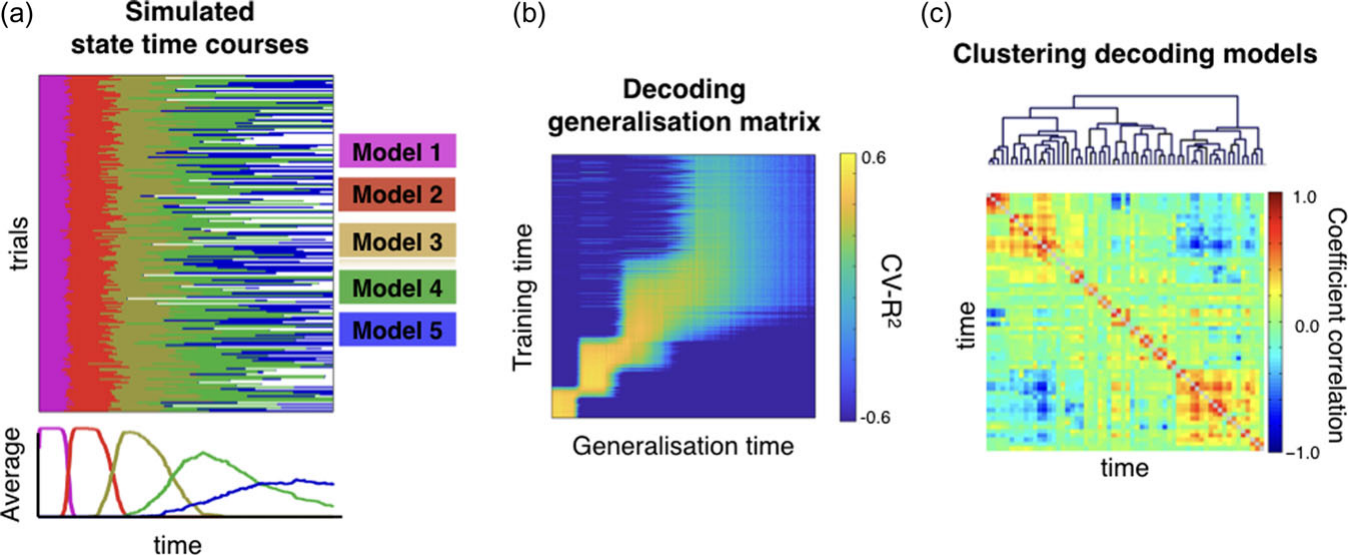
Failing to account for between-trial differences can result in a misleading view of stimulus processing. (*a*) Time-by-trial representation of which decoding model (of five) is active at each time point as simulated by a synthetic generative model (see Results); underneath, the across-trial average. (*b*) Cross-validation showing the generalization over time of the standard decoding approach (King and Dehaene 2014) suggests an artificial increasing of neuronal persistency as the trial progresses. (*c*) Hierarchical clustering of the estimated decoding models from the standard decoding approach suggests the presence of more than five distinct models (clustering is based on the correlation between their regression coefficients).

Figure 2*a* shows the simulated state time courses, where each color represents a different decoding model. Underneath, we show the across-trial average activation of each state or decoding model. Figure 2*b*,*c* illustrates the difficulties for the standard decoding approach in ignoring between-trial variability (and by using a larger number of decoding models than it exists). As is commonly done in the field when applying the standard decoding approach, Figure 2*b* shows the (cross-validated) generalization matrix, where, using cross-validated explained variance (CV-*R*^2^) as the summary statistic, we assessed the performance of each decoding model on held-out trials (King and Dehaene 2014). As observed, the trials generalize sharply at the beginning of the trials, since decoding model one is always active at start. Then, the pattern becomes blurrier and less accurate as a consequence of the increasing between-trial temporal variability. Standard interpretations of this result would artefactually suggest that brain activity gains in persistency (generalization) at the end of the trials, when, in reality, the models’ dwell time is the same across the entire trial. We also computed the Pearson correlation between sets of regression coefficients for each pair of models grouped the models according to their similarity. This is shown in Figure 2*c*, where we can see a large fragmentation into several different models, when, in reality, there are only five.****

### The Neural Processes Relevant to the Task Are Not Synchronous across Trials 

In this task, subjects were shown an oriented visual grating stimulus and asked to compare it to a memorized template orientation to detect matches (the difference between these being referred to as the “relative” angle). The model was estimated separately for each participant (of which there were ten) and each session (of which there were two per participant), obtaining a set of decoding models and model time courses indicating the probability of each model being active at each time point within each trial. We estimated the model for different numbers of decoding models (*K* = 3, 4, 5 and 6). More details about the method and the experimental paradigm are presented in Methods.

**Figure 3.**
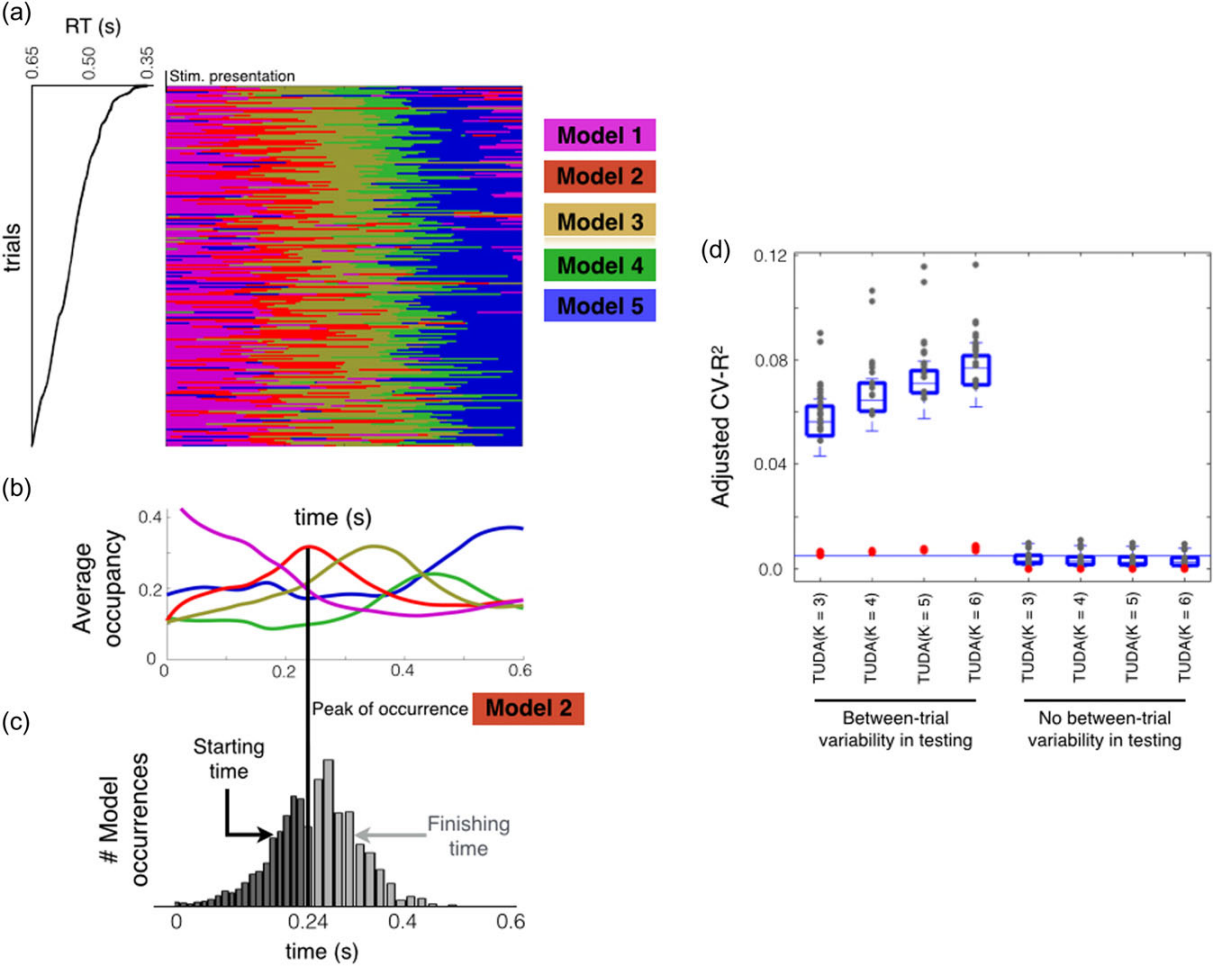
Decoding of the relative angle exhibits large temporal variability between trials. (*a*) Time-by-trial representation of which decoding model is active at each time point shown for a subset of the trials from a representative session and participant. Models are numbered according to the order in which they tend to arise in trials, and trials are ordered by RT; only 200 trials are shown. (*b*) Percentage of trials (taken over all subjects and trials) assigned to each decoding model as a function of time. (*c*) For a given decoding model, and this specific session, temporal variability represented as a histogram of starting and finishing times for those trials when the chosen decoding model is active at time *t* (*t* = 0.23 s for presented angle and *t* = 0.24 s for relative angle). (*d*) TUDA’s accuracy, measured as adjusted cross-validated explained variance (adjusted CV-*R*^2^, see Methods), when accounting for between-trial variability versus when between-trial differences are ignored, for different number of decoding models (*K* = 3, 4, 5 and 6). Each gray dot represents one session, the red dots represent the baseline accuracy obtained from surrogate data (see Methods), and the horizontal line represents (group level) cross-validated accuracy for the standard decoding approach.

Using a model with *K* = 5 decoding states on the real data (see Methods), Figure 3*a* shows when each of the five inferred decoding models (represented using a different color) is active as a function of time. This is shown for a subset of the trials ordered by RT. Note that the decoding is, by necessity, carried out separately for each session, so here we are presenting the results for a single participant and session. However, these results are representative of the results across the whole dataset (see Supplementary Material). Underneath, Figure 3*b* shows the average occupancy (i.e., the mean over all trials) of each decoding model as a function of time. We next show the extent of temporal variability of the decoding models for the proposed approach, that is, when we do not constrain the trials to have the same decoding dynamics. For this purpose, we chose one of the decoding models that had a clear peak in the average occupancy time course and took all trials where the model was active at the time of maximum model occupancy (represented by the marked peak in the bottom panel). In Figure 3*c*, histograms for the starting and finishing times of these model occurrences reveal large temporal variability.

We next investigated the extent to which accounting for between-trial temporal differences impacts the prediction accuracy of the model. Normally this would be estimated using cross-validation. However, TUDA uses both the data and the stimulus in combination to estimate the decoding model time courses (see Methods). This means that the stimulus information for a held-out trial gets used to estimate the decoding model time course for that trial (as well as for predicting the stimulus). We correct for this bias through the use of surrogate data, to calculate an adjusted CV-*R*^*2*^ measure (see Methods), allowing us to compare between different TUDA approaches and standard decoding.

Figure 3*d* shows adjusted CV-*R*^2^ for TUDA when we model between-trial temporal differences and when we do not, for different numbers of decoding models (*K*); that is, when no between-trial temporal differences are considered (four models on the right), meaning that we are having *K* instead of *T* decoding models, while still restricting the decoding models to be synchronous. As a reference, the horizontal line represents CV-*R*^2^ for standard decoding. The accuracy when modeling between-trial temporal differences is orders of magnitude larger than when ignoring such differences, highlighting the importance of these differences, and is also superior to the standard decoding approach despite using fewer models.

Finally, if between-trial temporal variability is such an important factor, we argue that, when ignoring this variability, the TUDA predictions should be significantly worse for those time points with greater diversity in decoding model allocation. For *K* = 5, Supplementary Figure 2*a* shows, for the same illustrative session used before, CV-*R*^2^ as a function of time for the traditional decoding approach and TUDA. Confirming this hypothesis, the peaks of accuracy closely correspond to the peaks of activation of the decoding models as shown underneath (where there are less between-trials temporal variability). Using all sessions, Supplementary Figure 2*b* shows, for each time point (represented as a dot), trial variability in the assignment of a decoding model in training (measured as the variance of the model time courses across trials) versus estimation accuracy in testing (measured using CV-*R*^2^); the Pearson correlation coefficient is 0.47 (*P*-value <0.0001, permutation testing).

### Sequences of States and Their Relation to Behavior 

If between-trial temporal variability has a neural origin, then we might also expect it to relate to behavior. Here, we show that this is the case and that the states follow very robust sequences but with varying dwelling time in each state across trials.

We analyzed RT data, which were collected for all trials where the subjects pressed the button (i.e., when participants judge that the presented angle matched the template angle in their working memory). We discarded all trials with no button press. Importantly, we first regressed the absolute relative angle out of the model time courses and the RTs. This is necessary because RT could have a direct correlation with the relative angle: smaller relative angles might make participants more confident, leading to faster responses. Without this precaution, a relation between the model time courses and RT could be trivially driven by the actual relative angle, instead of the intrinsic between-trial variability that is the focus of our work.

**Figure 4.**
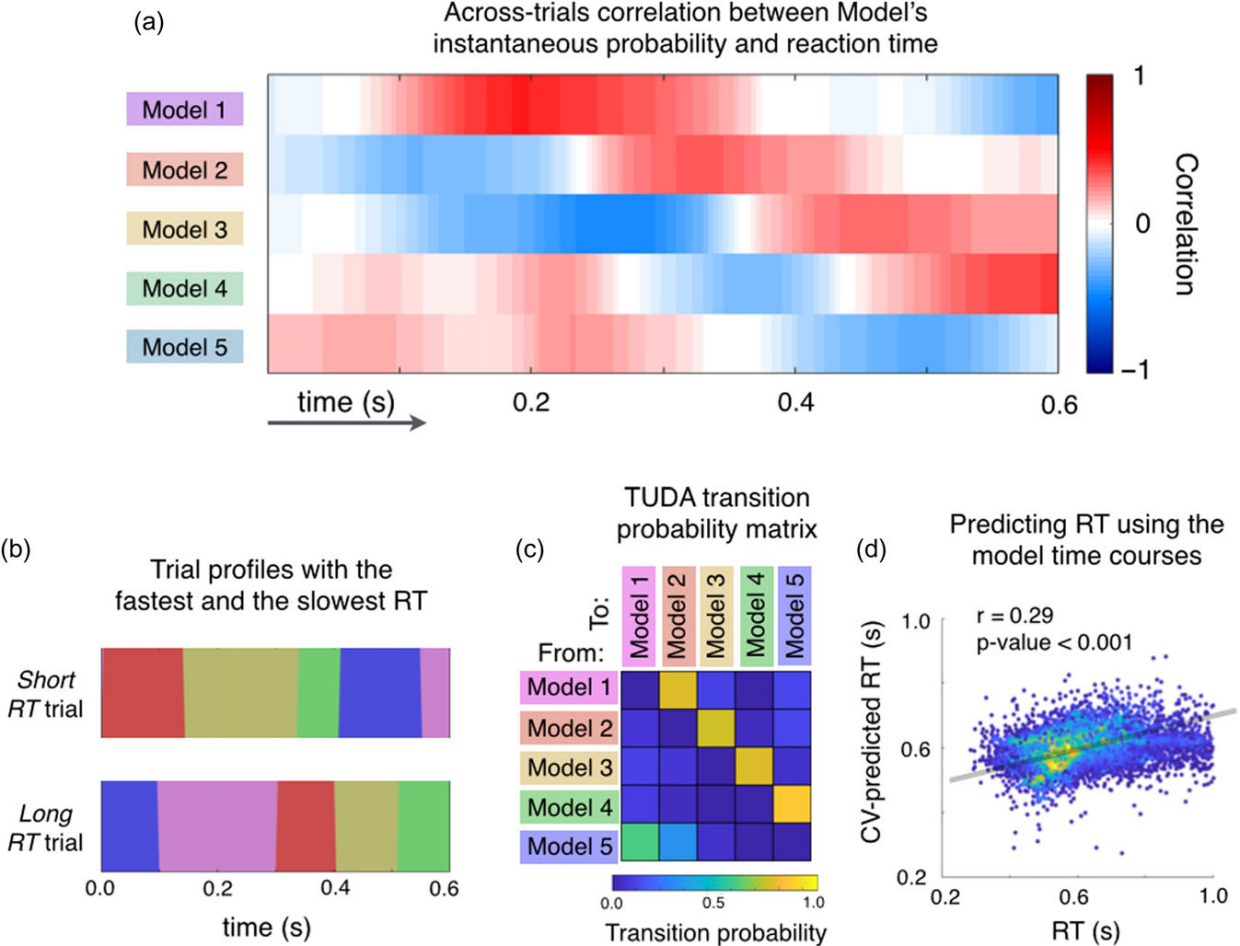
The precise timing of the decoding models within trials has an intimate relationship with RT. (*a*) For the same representative session used in Figure 3, the correlations between RT and each decoding models’ activation probabilities as a function of time, are very high. (*b*) Prototypical sequences of decoding models for a “short RT” trial (top) and “long RT” trial (bottom) for the representative session. (*c*) The transition probability matrix for the representative session, containing the probability of transitioning between each pair of models, has a strong sequential structure (see Supplementary Fig. 3 for other sessions). (*d*) Cross-validated prediction of RT as using the model time courses confirms the strong relationship between the models’ temporal variability and RT (each data point corresponds to one trial, and color indicates density of points). The plot depicts all sessions, although the prediction was performed session by session.

We then estimated, for each decoding model, the correlation between the corresponding (deconfounded) model time courses and RT across trials (i.e., the Pearson correlation between the probability of the state being active and RT). For an example session, Figure 4*a* shows the resulting time-resolved correlations for each of the decoding models, reflecting a strong relation between the decoding models’ trial-specific timings and behavior. On these grounds, we next examined how short RT trials compare to long RT trials, by viewing the temporal profile of which decoding model best predicts RT at each point in time. For an example session, Figure 4*b* shows these temporal profiles for prototypical “short RT” and “long RT” trials. The temporal profiles were calculated as follows: at each time point, the decoding model chosen to be active is the model with the highest across-trials correlation (for the short RT trial), or the highest anticorrelation (for the long RT trial), between the estimated probability of being active and RT. As can be seen, the same ordering of decoding models underlies short RT and long RT trials; however, the timing is very different, with the decoding models getting active around 0.25 s earlier in the short RT as compared to the long RT trials. As illustrated in Figure 4*c*, this characteristic sequence is also (separately) found in the transition probability matrix between the decoding models, which reflects the estimated probability of transitioning between every pair of decoding models, and is inferred without knowledge of RT as part of the model inference (see Methods). This strong sequential order of the decoding models is largely present in all participants and sessions (Supplementary Fig. 3).

We further evaluated the strength of the relationship between the model time dynamics and behavior by predicting, in a cross-validation setting, the trial RT using the model time courses (after regressing out the absolute relative angle from both variables; see Methods for details). The prediction was done at the group level, that is, using all participants together (cross-validation folds were constructed such that the entire set of trials of each subject were assigned to a single fold; Winkler et al. 2015). Figure 4*d* shows real versus predicted RT, where each dot represents a trial. The prediction accuracy is highly significant (*P*-value <0.001, permutation testing), confirming that the temporal decoding variability effectively relates to behavior. Note that a similar prediction would not be possible with the traditional approach, which does not provide any estimation of between-trial temporal variability.

### Decoding Models Are Spatially Localized

**Figure 5.**
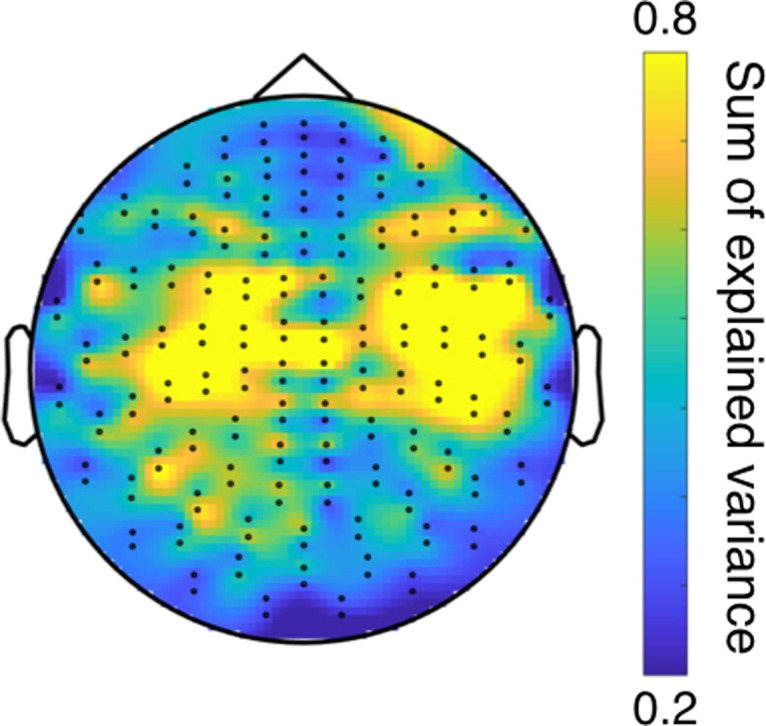
Topographical map in sensor space reflecting the (averaged) spatial activation associated to the estimated decoding models. This is expressed as the sum of explained variance (CV-*R*^2^) of the corresponding encoding models (see Methods).

We next examine the spatial characteristics of the decoding states. Interpretation of decoding weights is not straightforward (Weichwald et al. 2015), so we computed the “encoding” model that corresponds to each decoding model. For each sensor, decoding model and session, the encoding model is defined as the regression weights that predict the data for this sensor as a function of the relative angle, using only the time points when the decoding model is active (i.e., making use of the model time courses; see Methods). Figure 5 shows a summary of the spatial characteristics of the encoding models, which can be compared for reference with the maps shown in Myers et al. (2015). The topographic map represents the sum of explained variances across encoding models. Although there are differences across models and subjects, the maps indicate that the relative angle is encoded in motor and frontal sensors.

## Discussion

In this article, we use the Hidden Markov Modeling framework to propose a new approach capable of “time-resolved decoding,” referred to as TUDA. Using TUDA, it is possible to bypass the highly constraining assumption of consistent timing of states (or decoding models) across trials as made by the traditional decoding approach. By making decoding temporally unconstrained, we are able to gain new insight into the nature, sequence and temporal variability of neural states that contribute to the perceptual judgments made on different experimental trials.

An increasing number of studies have used time-specific pattern classification methods to show that stimulus-specific patterns are highly time specific and do not cross-generalize across time points. Furthermore, these methods suggest that neural processing traverses a large number of states in a smooth, stereotyped cascade (King and Dehaene 2014). This could occur through transitions from one attractor state to another within one circuit (Durstewitz et al. 2010; Miller 2016), or, similarly, through sudden transitions from a null state to a stimulus-specific state in a downstream brain area (Latimer et al. 2015). However, this trajectory through neural state space assumes that trajectories are highly reproducible across trials. By relaxing this assumption, we found that processing might possibly have slower dynamics than suggested by the standard decoding approach. Importantly, we found these dynamics to be related to behavior, exemplified here as RT.

The advantages of TUDA are best illustrated by comparing the results we present here to those reported in a previous analysis of the same dataset (Myers et al. 2015). In this previous study, we also found reliable and strong time-specific decoding of the decision variable using the conventional cross-temporal generalization approach. In addition, while cross-temporal generalization was weaker than within-time decoding, it was nevertheless highly significant, indicating both dynamic and time-stable decoding. Using conventional cross-temporal generalization, the result was ambiguous and could be explained in various ways. For example, such a pattern could be the result of one neural population encoding the decision variable in a dynamic code and a second population encoding it in a static code. The present results suggest instead a simpler interpretation: a single pattern encoded the decision variable, but temporal variability in its onset across trials leads to cross-temporal generalization.

Other studies have found similar patterns of increasingly reliable cross-temporal generalization at later time points in a trial, which have sometimes been interpreted as evidence of time-stable coding in delay periods after initial stimulus processing is complete (e.g., Stokes et al. 2013). Again, the current findings suggest that, to some extent, the appearance of temporally stable patterns could instead be the result of increasing temporal variability in the onset of the same coding pattern across trials. More generally, using TUDA to evaluate the temporal variability versus persistence of states could help resolve an ongoing debate concerning persistent (Constantinidis et al. 2018) versus intermittent but trial-variable coding (Lundqvist et al. 2018) of working memory content during maintenance delays.

Furthermore, the finding of highly dynamic coding in our initial study (Myers et al. 2015) raised a conceptual difficulty: how could downstream areas make sense of a highly time-varying code? In order to readout the stimulus encoded in such a dynamic pattern, the pattern of synaptic strengths onto the downstream population would need to evolve as dynamically as the code itself to ensure constant and accurate readout. Using TUDA, this problem is resolved by the finding that only a small number of time-variable states explain the data: since decoding patterns are stable for the duration of each state, downstream areas will only need a single set of weights to read out the stimulus encoded during that state. Therefore, there is no need to resort to dynamically changing synapses.

Finally, TUDA explicitly models the onset times of states on each trial, making it possible to link neural timing to behavioral timing (e.g., RTs). Testing for such a brain–behavior correlation is not straightforward using the conventional decoding framework. Using TUDA, the estimated between-trial temporal differences can be further related to changes in attention and also learning and plasticity.

### Alternative Decoding Models

Here, we have used the instantaneous (raw) sensor-space MEG signal to predict the stimulus, in analogy to the study from Myers et al. (2015) based on the same dataset. Both Myers and colleagues’ approach and ours use information about the relative magnitude between sensors in order to decode and disregard other information. Given that the stimulus at time point *t* is predicted using only data at time point *t*, this approach is, for example, blind to the oscillations that encompass the instantaneous signal used for the prediction.

More powerful (or interpretable) extensions, where the phase of ongoing oscillations is used for the prediction, are straightforward to implement under the proposed HMM-based framework. For instance, similar to Vidaurre et al. (2018), the signal can be “embedded” such that a window around *t* (and not only *t*) is used to predict the stimulus at time *t*, effectively incorporating phase information into the estimation. Other possibilities are to use information of phase only (Cabral et al. 2017) or power only (Baker et al. 2014). Unlike the embedded approach, which uses the raw signal without the need of any mathematical transformation, these alternatives are based on the Hilbert transform and the use of filtering, for which an ad hoc selection of the frequency bands of interest is required. Besides the decision of which features of the data will constitute the base for the prediction, another possible extension is to replace the simple, linear regression model by more powerful prediction algorithms such as support vector machines or neural networks (Hastie et al. 2001) as far as these are formulated within the Bayesian framework. This scenario, where the decoding models may have a much larger number of parameters, can easily be handled with the proposed framework (where there are only *K* models) but is less manageable for the standard approach (where there are *T* models), especially if the number of trials is not very large.

### Decoding with fMRI 

In this work, we found temporal variability between trials in the range of hundreds of milliseconds. Although this is a significant amount of time when considering electrophysiological data, fMRI has much lower temporal resolution and the temporal uncertainty brought about by the hemodynamic response further hampers the benefits of our approach for probing tasks with fast cognitive mechanisms (attention, perception, etc.). There are however tasks with meaningful variability at the fMRI scale (seconds rather than milliseconds): difficult decision making, mind-wandering, tasks with components that fluctuate slowly such as arousal and tasks with delayed activity such as those related to working memory. In these types of tasks, the proposed method has the potential to excel at discovering temporal variability, up to the limit imposed by the modality’s inherent temporal resolution and its haemodynamics.

### Localized Decoders 

Here, we applied the model to whole-brain sensor space data, in line with previous work (Myers et al. 2015). Finer spatial and temporal information can be obtained from applying this method to source-localized data, possibly running the model on one group of regions at a time. This approach can give us insight on the different temporal dynamics of various regions in encoding the stimulus by examining and post hoc comparing the model time courses between regions. This strategy would be comparable to the analyses performed by Baldassano et al. (2017) with an unsupervised HMM (i.e., trained with no information of the task), where sequences of states where estimated from different brain regions while subjects watched movie-based stimuli. This study revealed that higher order regions follow state segmentations that match the movie structure more closely than those followed by sensory regions. By including the stimulus (or certain aspects of it) into the model, we can however target more specific aspects of cognition and will benefit from higher sensitivity.

### Null States

The HMM is a general framework that has been used previously to describe brain activity in an unsupervised fashion (see, e.g., Baker et al. 2014; Engel et al. 2016; Baldassano et al. 2017; Vidaurre et al. 2016, 2018; Vidaurre, Abeysuriya, et al. 2017; Vidaurre, Smith, et al. 2017). Here, we draw from this general framework to handle the supervised setting, where each HMM state corresponds to a certain particular relationship between bran activity and the stimuli, but what happens for these time points where there is no relationship between brain activity and the stimulus at all (for instance, because the brain has not yet encoded the information in any way)? In an unsupervised setting, because the brain is never silent, all states are always meaningful (they always represent something, because there is brain activity in all time points). In a supervised setting, however, it is useful to detect when there is “nothing” to represent. In the current implementation, the proposed model can express this circumstance by using a “null” state (where the decoding weights are close to zero), or, instead, by using some random mixture of (otherwise meaningful) states such that, at these “empty” time points, the decoding error is higher than when these states are faithfully representing the stimulus. Post hoc analyses would be required to detect this situation. A potentially more useful strategy would be to fix one decoding state to be the null state (i.e., having fixed, zeroed decoding weights), such that it will become active when the examined regions are unaware of the stimulus. In the data used in this article, nevertheless, this would likely be of little use given the short inter-stimuli intervals.

## Conclusion

In this article, we proposed a novel method for neural decoding, referred to as TUDA, where we dispense altogether with the assumption that neural processing is timed consistently across trials. Our results, on a simple perceptual decision-making task, indeed suggest that the assumption of consistent timing over trials made by a traditional decoding approach is not always justified and can lead to misinterpretations of the dynamics of the cognitive mechanisms underpinning the processing of the stimulus. Although we have focused on a relatively simple stimulus, the technique can straightforwardly be applied to more complex cognitive tasks including volitional behavior. Our approach also makes it possible to analyze the between-trial temporal variability, which, as shown earlier, can hold a significant relationship to behavior and could correspond to changes in attention or to plasticity.

## Supplementary Material

Supplementary Data
