# Supplementary material for: Temporally Unconstrained Decoding Reveals Consistent but Time-Varying Stages of Stimulus Processing
Source: Cereb Cortex. 2018 Dec 7;29(2):863–74. doi: 10.1093/cercor/bhy290 (PMC6319313; doi:10.1093/cercor/bhy290)
Supplement: Supplementary Data [file bhy290_vidaurre_si_cc_r1.docx]

**Supplemental Information**


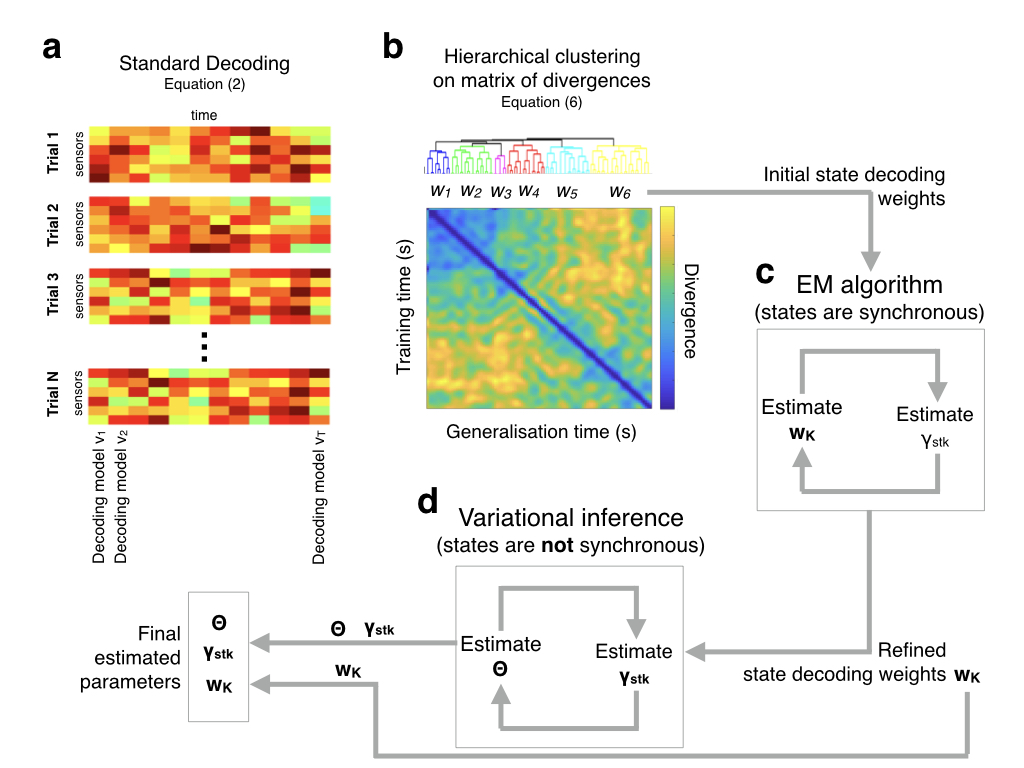


**Figure SI-1**. Schematic of the parameter inference, which comprises the following steps: (**a**) we perform standard decoding; (**b**) we use hierarchical clustering, grouping the obtained decoding models into *K* clusters; (**c**) using this as an initial temporal asignment of when the states are active, and while still assuming the states to be synchronous across trials, we run the expectation-maximisation (EM) algorithm to estimate the decoding coefficients; (**d**) using these decoding coefficients and dispensing with the assumption of between-trial synchrony, we use Bayesian variational inference to finally estimate the final sequence of states and the between-states transition probabilities.


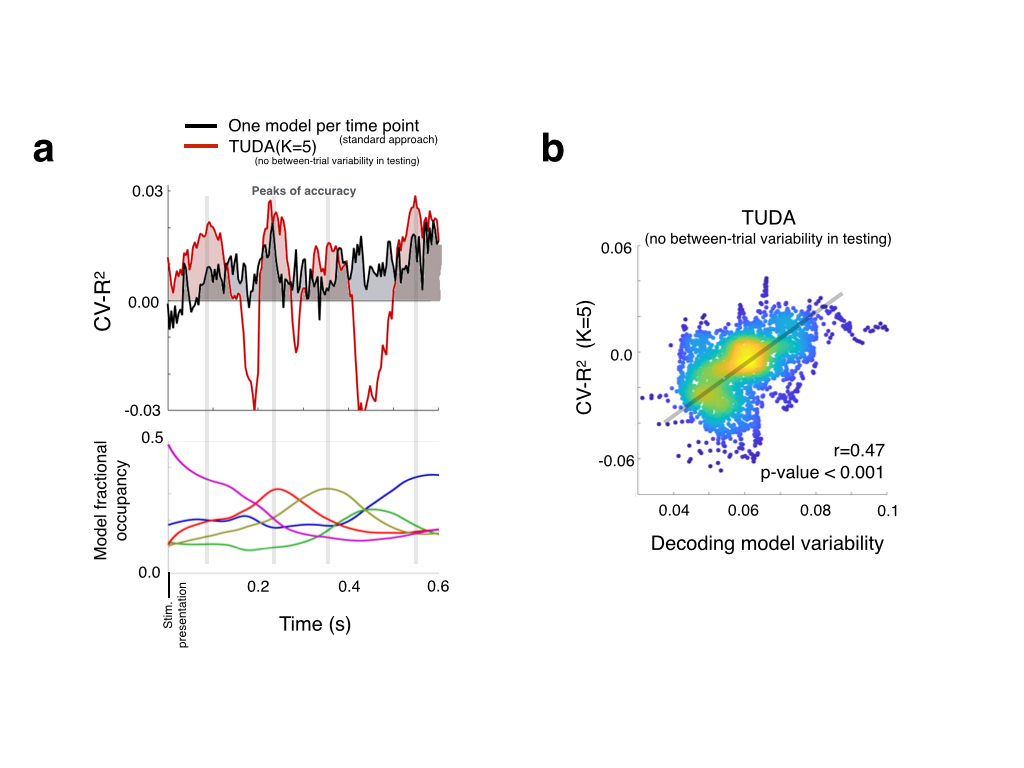


**Figure SI-2**. Cross-validation underestimates TUDA’s performance if we lose between-trials temporal variability in the held-out trials. (**a**) For the same illustrative session used in Figure 2, CV-R^2^ is shown as a function of time for the standard approach (black) and the proposed model when using *K*=5 decoding models (red). Underneath, the model fractional occupancy (see Figure 2) reveals that the peaks of accuracy closely correspond to the time points with less between-trials temporal variability. (**b**) There is a strong correlation between decoding model uncertainty (as expressed by the variability of the model time courses across trials) and accuracy (as expressed by the cross-validated explained variance, CV-R^2^), where each data-point in the scatter plot corresponds to a time-point within the trial (colour represents density of points and the line represents the slope of regressing model variability on CV-R^2^).


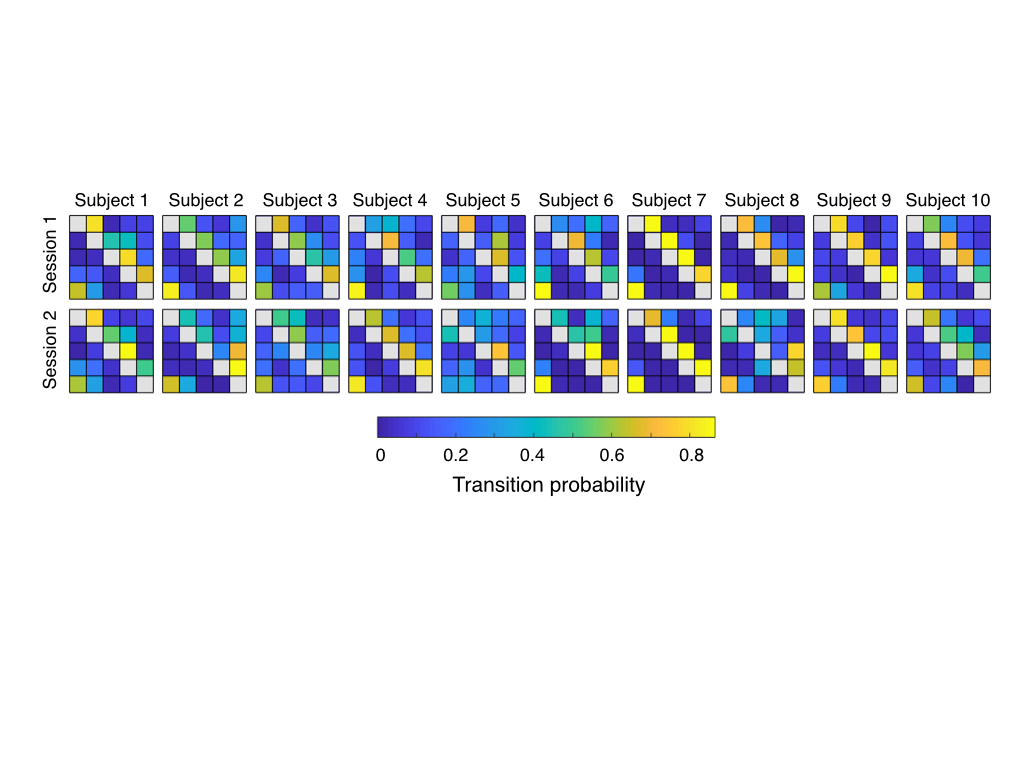


**Figure SI-3**. Transition probability matrices between decoding models for all sessions and subjects reveal that information processing follows consistent sequences of states in most of the sessions.
